# Pathway-Based Genetic Risk Scores Are Associated with Blood Lipids Among Young Mexican Adults

**DOI:** 10.3390/nu18060979

**Published:** 2026-03-19

**Authors:** Bridget A. Hannon Esteves, Margarita Teran-Garcia, Flavia C. D. Andrade, Itzel Vázquez-Vidal, Juan Manuel Vargas-Morales, Celia Aradillas-Garcia

**Affiliations:** 1 Division of Nutritional Sciences, University of Illinois Urbana-Champaign, Urbana, IL 61801, USA; 2 Illinois Extension, College of Agricultural, Consumer and Environmental Sciences, University of Illinois Urbana-Champaign, Urbana, IL 61801, USA; 3 Carle Illinois College of Medicine, University of Illinois Urbana-Champaign, Urbana, IL 61801, USA; 4 Department of Food Sciences and Human Nutrition, University of Illinois Urbana-Champaign, Urbana, IL 61801, USA; itzel.vazquezvidal@umanitoba.ca; 5 School of Social Work, University of Illinois Urbana-Champaign, Urbana, IL 61801, USA; fandrade@illinois.edu; 6 Richardson Centre for Functional Foods and Nutraceuticals, University of Manitoba, Winnipeg, MB R3E 0W2, Canada; 7 Laboratorio de Análisis Clínicos, Facultad de Ciencias Químicas, Universidad Autónoma de San Luis Potosí, San Luis Potosí 78000, Mexico; juan.vargas@uaslp.mx; 8 Centro de Investigación Aplicada en Ambiente y Salud, CIACyT, Universidad Autónoma de San Luis Potosí, San Luis Potosí 78000, Mexico; 9 Facultad de Medicina, Universidad Autónoma de San Luis Potosí, San Luis Potosí 78000, Mexico

**Keywords:** dyslipidemia, genetics, SNP, cardiovascular risk, genetic risk score

## Abstract

**Background/Objectives:** Mexicans are disproportionately affected by dyslipidemia, specifically low high-density lipoprotein (HDL-C) and high triglyceride (TG) concentrations. Research on the genetic contributions to dyslipidemia, conducted primarily among European populations, has identified numerous single-nucleotide polymorphisms (SNPs) with small effect sizes and low replication rates. A genetic risk score (GRS) can examine the cumulative effects of multiple SNPs and potentially explain greater phenotypic variability than individual SNPs. GRS in Mexican populations and those without diagnosed dyslipidemia are limited. This study aims to construct a GRS from lipid metabolism-related SNPs and determine its associations with blood lipid concentrations in young Mexican college students. **Methods:** Adults (ages 18–25 years, *n* = 580) provided a fasting blood sample to determine TG and HDL-C concentrations. DNA was genotyped for 14 SNPs in lipid metabolism pathways (reverse cholesterol transport [RCT], cellular lipid uptake, and lipoprotein formation and transport). Additive (number of risk alleles) and weighted (regression-derived β coefficients) GRS were calculated for individual pathways, and their sum (total GRS) was explored. Associations among individual SNPs, GRS, and blood lipids were determined through general linear models in SAS. **Results:** The additive RCT and total GRS were associated with TG (both *p* < 0.05). The RCT pathway explained 3.4% of the variability in TG concentrations, and the total GRS explained 6.1%. The weighted RCT GRS was associated with HDL-C (*p* = 0.007). The ATP-binding cassette protein (ABCA1) rs9282541 variant was most strongly associated with HDL-C (*p* = 0.016). When this SNP was removed from the GRS, the association became non-significant. **Conclusions:** SNPs in lipoprotein metabolism pathways cumulatively associate with blood lipid concentrations in young Mexican adults. The ABCA1-rs9282541 variant, previously shown to be positively associated with low HDL-C concentrations in Amerindian populations, had the strongest association with HDL-C. Further work is needed to elucidate the roles of genetic admixture and lifestyle risk factors in dyslipidemia in this population.

## 1. Introduction

The Mexican population is disproportionally affected by dyslipidemia, due to a combination of genetic predisposition and lifestyle (i.e., diet and physical activity) factors [1]. Common dyslipidemias in this population are low high-density lipoprotein cholesterol (HDL-C) and high triglyceride (TG) concentrations, affecting 55% and 47% of the population, respectively [2]. These phenotypes are also included as criteria for the Metabolic Syndrome, a cluster of obesity-related risk factors associated with type 2 diabetes mellitus (T2DM) and cardiovascular disease (CVD), two of the leading causes of death in Mexico [3]. Despite the high prevalence of dyslipidemia and the significant genetic component to this disease, there has been considerably less research conducted among young Mexicans, who also have a high prevalence of low HDL-C (52%) and high TG (32%) concentrations. Genetic predisposition to dyslipidemia accounts for approximately 70% of the variability in elevated blood lipid concentrations [4]. Over 150 loci have been associated with blood lipid concentrations (total cholesterol, TG, HDL-C, and low-density lipoprotein cholesterol) in European populations [5,6]. However, genetic contributions to dyslipidemia vary greatly across populations, and approximately 96% of gene association studies have been conducted among individuals of European descent, demonstrating the lack of diversity in genetic research, especially among at-risk populations such as Mexicans [7].

A recent genome-wide association study (GWAS) conducted in Mexicans identified novel loci associated with TG concentrations [8]. These loci, in the form of single-nucleotide polymorphisms (SNPs), are common in the population and do not exert strong effects on blood lipid phenotypes when considered alone. In most individuals, the genetic contribution to dyslipidemia is the result of multiple, common SNPs exerting minor effects. Still, several common SNPs have been evidenced to be selectively associated with dyslipidemia in Amerindian and Mexican populations. Examples include the rs9282541 variant of ATP-binding cassette protein member 1 (ABCA1), more common among Amerindian and Mexican populations than Europeans, and the rs805743 intergenic locus, which was recently discovered through GWAS to associate with TG concentrations [8,9]. However, the presence of these variants alone will not result in dyslipidemia. Due to the polygenic nature of this disease, studying multiple variants collectively, such as in a genetic risk score (GRS), may provide a greater explanation of phenotypic variability than investigating individual SNPs.

GRS have been previously constructed to determine associations between common variants and the incidence of T2DM [10,11], and various CVD-related traits [12,13,14]. The inclusion of SNPs in a GRS is usually determined by the strength of the SNP’s association with the phenotype in previously published literature. However, the “top hits” approach does not always account for the biological plausibility of the selected SNPs. Some GRS are constructed with consideration of the physiological relevance, for example, a pathway-based GRS [15]. In the pathway approach, variants are included in the GRS if they have been identified as associated with the trait of interest and are in pathways relevant to that trait or disease progression (e.g., vasodilatory pathway genes and hypertension risk) [16]. The significance of a pathway-based GRS lies in the functional, rather than merely associative, relationships between SNPs and the phenotype. GRS can be constructed as additive, meaning the score represents an individual’s number of risk alleles, which allows all variants to contribute equally to risk [17], or weighted, which allows for variants with a stronger association to contribute more to the overall score [18,19]. Studies employing a weighted approach have often obtained weights from previous genetic work to reduce bias and model overfitting [18]. However, effect sizes for many SNPs in understudied populations, such as Mexicans, are not always available [13,20].

This study seeks to determine associations between individual SNPs previously associated with blood lipid concentrations and to explore the cumulative risk associated with additive and weighted GRS for blood lipid concentrations in young Mexican adults.

## 2. Materials and Methods

### 2.1. Study Design and Populations

Young Mexican college students (aged 18–25 years, *n* = 580) from the UP-AMIGOS Cohort study (Universities of San Luis Potosí and Illinois: A Multidisciplinary Investigation on Genetics, Obesity, and Social-environment) were included in this analysis [21]. Participants were college applicants to the Autonomous University of San Luis Potosí in Mexico. The Institutional Review Boards of both universities approved all study protocols. Participants provided informed consent prior to any study procedures. Height and weight were measured in light clothing by trained research staff. BMI was calculated as kg/m^2^. A venous blood sample was collected after an overnight fast for biomarker and genetic analysis. HDL-C and TG concentrations were measured using colorimetric enzymatic reactions.

### 2.2. SNP Selection

Genomic DNA was extracted from whole blood (Gentra Puregene Blood Kit, Qiagen, Valencia, CA, USA), and genotyped at the W.M. Keck Center (University of Illinois Urbana-Champaign) using the Fluidigm SNP genotyping platform (Fluidigm Corp., San Francisco, CA, USA). Genotypes were called using the Fluidigm Genotyping Analysis Software v4.1.2 platform (Fluidigm Corp., San Francisco, CA, USA), with a minimum confidence level of 85%.

Fourteen common variants from genes involved in pathways related to lipoprotein metabolism were selected from the NCBI database (https://www.ncbi.nlm.nih.gov/snp) based on their previous associations with blood lipid traits and classification as functional variants (missense, intronic, etc.) or those that have been shown to affect transcription. The STRING database (https://string-db.org) was then used to organize the selected variants into physiologically relevant pathways. Three pathways relevant to lipid and lipoprotein metabolism were identified for these analyses: reverse cholesterol transport (RCT), cellular lipid uptake (CLU), and lipoprotein formation (LPF) (Table 1). Samples were also genotyped for 64 ancestral informative markers (AIMs) that were used to create a principal component score to adjust for the genetic admixture of the population [22].

### 2.3. GRS Construction

Two GRS were constructed to explore and assess cumulative genetic risk: additive and weighted [19]. In the additive GRS, each genotype was assigned a value of 0, 1, or 2, corresponding to the number of risk alleles present in this sample, with a maximum combined GRS expected of 28 (i.e., a homozygote for all 14 SNPs). The risk allele was determined by evaluating the least-squares means (LS means) for TG and HDL-C. All additive GRS were calculated as the sum of the risk alleles present in an individual for the variants in each of the three pathways (RCT, CLU, or LPF) and the “top hits” SNPs pathway. In the weighted GRS, estimates of the beta coefficient obtained from individual SNP associations were used to weigh each SNP by its effect size (β estimate). GRS were constructed for each of the 3 pathways and the “top hits” SNPs. High or low genetic risk was determined by using the median value to split the sample in 2 groups [23].

### 2.4. Statistical Analysis

All variables were assessed for normality; non-normal variables were logarithmically transformed for analysis and then back-transformed for interpretation. Student’s *t*-tests were used to assess significant differences in variables of interest by sex. Participant characteristics are presented as mean ± standard error (SE). Minor allele frequencies (MAF) and Hardy–Weinberg Equilibrium (HWE) were calculated in SAS v9.4 (Cary, NC, USA).

Deviations from HWE were assessed using χ^2^ test. SNPs that were not in HWE were excluded from further analyses. SNP-SNP interactions for associations with both phenotypes were determined using an interaction term in the model. Linkage disequilibrium (LD) among variants within a gene or on the same chromosome was assessed using LDlink (https://ldlink.nci.nih.gov) with the 1000 Genomes MXL population as the reference. If variants were found to be in LD, the SNP that explained the greatest variance in the phenotype was used in the exploratory GRS construction.

Associations between TG and HDL-C concentrations and individual SNPs were assessed using general linear models (GLMs) adjusted for age and sex. Models were also analyzed with BMI as a covariate to adjust for adiposity status. Following individual associations, similar models were used to detect associations with genetic risk (RCT, CLU, LFP, and cumulative GRS). Significant differences in TG and HDL-C concentrations between individuals at high vs. low genetic risk were determined by Student’s *t*-test. The Tukey–Kramer adjustment for multiple testing was applied in all GLMs. A *p* < 0.05 was considered statistically significant, and a *p* < 0.10 was approaching statistical significance.

Exploratory analyses were conducted to assess differences in phenotypic variability explained by SNPs with strong statistical associations versus those with biological relevance. A “top-hits” GRS was constructed from only SNPs that were individually associated with the HDL-C or TG phenotype at *p* < 0.05 (statistical significance) and *p* < 0.10 (approaching statistical significance). Models were adjusted first for age and sex, and then BMI was added. Next, to determine the statistical effect of the most strongly associated SNP (key SNP) for each phenotype, GRSs were reconstructed excluding that SNP. Finally, significant SNP-SNP interactions for each phenotype were added to the GRS to explore potential genetic interactions. These analyses were also conducted with the AIMs principal component scores as covariates. The first two principal component scores accounted for 5% of the population variance and were sufficient for analysis, as determined by factor analysis.

## 3. Results

### 3.1. Sample Characteristics

Demographic characteristics are presented in Table 2. A total of 580 individuals (265 males, 315 females) participated in this study. The population sample had a low prevalence of overweight individuals (17%, *n* = 99), obesity (13%, *n* = 75), and hypertriglyceridemia (16%, *n* = 93). However, almost half (49%) of individuals had low HDL-C concentrations. Compared to females, males had a higher BMI and lower HDL-C concentrations (*p* = 0.01 and *p* < 0.01, respectively).

### 3.2. Linkage Disequilibrium and SNP-SNP Interactions

The genotype distribution for all included SNPs, except CD36-rs3173798, was in HWE. Two SNPs in ABCA1 (rs9282541 and rs4149310) showed high LD (D′ = 1.0, R^2^ = 0.041). All three SNPs in ANGPTL4 were also in high LD. Since ANGPTL4-rs2278236 explained greater variability in both phenotypes, it was selected as the tag SNP for ANGPTL4. Additionally, CD36-rs10499859 and CD36-rs1527483 exhibited high LD (D’ = 1.0, R^2^ = 0.243), with the former included in the GRS.

Several SNP-SNP interactions associated with TG concentrations were identified, including LOC-rs805743 with CETP-rs289714 (*p* = 0.007), and CETP-rs5882 with ABCA1-rs4149310 (*p* = 0.039). The interaction between LIPC-1800588 and MLXIPL-rs2286276 (*p* = 0.039) was significantly associated with TG levels. Additionally, notable interactions were observed between ABCA1-rs9282541 and two CETP SNPs: rs289714 (*p* = 0.042) and rs5882 (*p* = 0.002), both of which were associated with HDL-C concentrations. The interaction between CD36-rs10499859 and LPL-rs12678919 was significantly associated with HDL-C (*p* = 0.009).

### 3.3. Individual SNP Associations

Individual SNP associations with lipid profile are presented in Supplementary Table S1 (TG) and Table S2 (HDL-C). The following SNPs were associated with TG concentrations: CETP-rs1532624 (*p* = 0.0442, R^2^ =0.013) and MLXIPL-rs2886276 (*p* = 0.0023, R^2^ = 0.009). The rs9282541 variant of ABCA1 was associated with HDL-C concentrations (*p* = 0.0157, SNP R^2^ = 0.0161).

### 3.4. Genetic Risk Scores

From the three pathways identified in the SNP selection, 14 SNPs were included in GRS construction. In several SNPs, the risk allele differed between TG and HDL-C phenotypes. Therefore, separate GRS were constructed. For TG, the following SNPs were included: RCT Pathway: LOC-rs805743, CETP-rs1532624, CETP-rs289714, CETP-rs5882, ABCA1-rs4149310; CLU Pathway: ANGPTL3-rs10889337, ANGPTL4-rs2278236, CD36-rs10499859, LPL-rs12678919; LPF Pathway: GCKR-rs1260326, LIPC-rs1800588, MLXIPL-rs2886276, PPARG-rs1801282, and PPARG-rs12639162. The “top hits” GRS for TG included: CETP-rs5882, CETP-rs1532624, ABCA1-rs4149310, MLXIPL-rs2886276, LIPC-rs1800588, and PPARG-rs12639162. For HDL-C, the following SNPs were included: RCT Pathway: LOC-rs805743, CETP-rs1532624, CETP-rs289714, CETP-rs5882, ABCA1-rs9282541; CLU Pathway: ANGPTL3-rs10889337, ANGPTL4-rs2278236, CD36-rs10499859, and LPL-rs12678919; LPF Pathway: GCKR-rs1260326, LIPC-rs1800588, MLXIPL-rs2886276, PPARG-rs1801282, and PPARG-rs12639162. The “top hits” GRS for HDL-C included CETP-rs289714, ABCA1-rs9282541, and LIPC-rs1800588.

There were also significant differences in TG concentrations between those at high risk (more than 15 alleles in the additive total GRS) and those at low risk. Individuals at high genetic risk had a mean TG concentration of 104.9 ± 2.5 mg/dL, whereas those at low risk had a mean TG concentration of 95.5 ± 1.9 mg/dL (*p* = 0.003). No significant differences in HDL-C concentrations were observed. The differences in blood lipids by risk category are presented in Supplementary Table S3. In the additive total GRS, the average score (number of risk alleles) was 15.3 ± 2.6 for TG, with a maximum expected score of 28 (i.e., an individual homozygous for the risk allele at all 14 SNPs). The average weighted total GRS was 0.2 ± 0.1 for TG and -11.0 ± 5.5 for HDL-C concentrations. The median scores for the total GRS and each pathway are presented in Supplementary Table S4. The beta coefficients for individual SNPs and the two phenotypes used to calculate the weighted GRS are presented in Supplementary Table S5.

### 3.5. Associations Between Genetic Risk and Blood Lipids

Associations between GRSs and blood lipids are presented in Table 3. The additive RCT pathway was significantly associated with TG concentrations (R^2^ = 0.050, GRS *p* = 0.0017), as was the additive total GRS (R^2^ = 0.071, GRS *p* = 0.0114). Estimates of the beta coefficients for both the RCT pathway (3.59, 95% confidence interval [CI]: 1.10, 6.04), and the cumulative GRS (2.35, 95% CI: 0.65, 4.04) were statistically significant. The RCT-GRS pathway accounted for 3.4% of the variation in TG concentrations, and the total GRS explained 6.1%. No significant associations between HDL-C concentrations and any additive GRS were found. The weighted CLU pathway was associated with TG concentrations (R^2^ = 0.167; GRS *p* = 0.0067), and genetic risk explained 14.4% of the variability in TG concentrations. The weighted RCT and the weighted cumulative GRS were associated with HDL-C concentrations (R^2^ = 0.314, GRS *p* = 0.0032; R^2^ = 0.040, GRS *p* = 0.0339), explaining 20.2% and 1.0% of the variability in HDL-C concentrations, respectively. When analyzed separately by sex, significant associations were found only among males. The weighted RCT pathway showed a significant association with HDL-C in males (R^2^ = 0.565, *p* = 0.0069), as did the weighted CLU pathway (R^2^ = 0.546, *p* = 0.0314).

### 3.6. Role of BMI

All associations remained when BMI was added as a covariate in the model (Table 4). The additive RCT pathway was significantly associated with TG concentrations (R^2^ = 0.193, GRS *p* = 0.0144), as was the additive cumulative GRS (R^2^ = 0.212, GRS *p* = 0.0168). The RCT pathway explained 3.3% of the variability in TG concentrations, and the cumulative GRS explained 4.9%. There were no significant associations between HDL-C concentrations and any additive risk scores. Regarding the weighted scores, only the weighted CLU pathway was associated with TG concentrations (R^2^ = 0.265, GRS *p* = 0.0049), and genetic risk accounted for 12.7% of the variability in TG concentrations. The weighted RCT and the weighted cumulative GRS were both significantly associated with HDL-C concentrations (R^2^ = 0.330, GRS *p* = 0.0057; R^2^ = 0.146, GRS *p* = 0.0191), explaining 24.7% and 1.0% of the variability in HDL-C concentrations, respectively.

### 3.7. Role of Ancestry

Genetic admixture in the sample was accounted for by including the AIMs principal component score as a covariate in all models [24]. No significant associations with GRS were detected when AIMs were included in the model.

## 4. Discussion

This study provides new insights into genetic factors influencing dyslipidemia in healthy young Mexican adults. Variants that are physiologically significant in pathways involved in lipid and lipoprotein metabolism are collectively associated with TG levels through an exploratory additive genetic risk score (GRS). In the exploratory weighted GRS, significant associations were identified for HDL-C concentrations. Both phenotypes showed strong associations with the RCT pathway, primarily driven by variants in CETP and ABCA1. These SNPs have been previously associated with blood lipid concentrations in this population. The Mexican population is unique because it is genetically admixed with European, African, and Amerindian variants; therefore, ancestry was included in the analyses. However, no effects were observed from including genetic admixture in the statistical models. The population-specific associated variants may have large enough effect sizes to be significant with or without accounting for genetic admixture.

Previous research by our group and others has shown that the minor allele frequencies (MAFs) of several lipid-metabolism-related SNPs differ in the Mexican population, suggesting that further research is needed to better understand the genetic factors contributing to dyslipidemia in this high-risk group [13,17,20,24,25,26,27,28,29,30]. The specific associations identified here, particularly those involving SNPs in MLXIPL, LIPC, and ABCA1, have been previously reported in Mexicans [8]. For instance, MLXIPL-rs2286276 is associated with TG concentrations in both Mexican and European populations [8]. LIPC-rs1800588 has been associated with hypertriglyceridemia and an increased risk of type 2 diabetes in a case–control study of Mexican Mestizos with coronary artery disease [26,31]. ABCA1-rs9282541 is a population-specific gene variant consistently associated with low HDL-C in Mexican and Amerindian populations [9]. Since each association accounts for only a small portion of the phenotypic variation, an experimental GRS approach was explored in this report.

In the post-GWAS era, the combined effects of genetic loci related to lipid metabolism on blood lipid phenotypes have been studied before using both additive and weighted GRS, with mixed results [32,33]. An additive method was used to evaluate genetic contributions to how weight loss affects blood lipid levels, using SNPs previously associated in GWAS [30]. In this study, the risk allele was selected based on its association with the undesirable phenotype in the UP-AMIGOS Cohort, noting that allele frequencies differ by ethnicity and that data on the SNPs included in non-European populations are limited [7]. The study also applied a weighted method to explore the strength of individual variants’ associations. Past research indicates that a weighted score explains more variation in the phenotype than an additive score [18,19]. Studies using a weighted approach often derive weights from prior genetic research to minimize bias and overfitting [18]. This method is only feasible when all cohorts share the same ethnicity, which was not the case here; therefore, the effect sizes were calculated from the current sample. Currently, PGS are used as statistical predictors rather than diagnostic tools. Individuals with high scores may never develop the phenotype, while those with low scores might. Interpretation should always consider the population context, the definition of the phenotype, and the validation results [32,33,34,35]. Overall, the UPAMIGOS Cohort aims to identify individuals at early risk of metabolic diseases, including dyslipidemia, to encourage lifestyle and behavioral changes and prevent adverse health outcomes.

Our analysis showed that the GRS based solely on the top-associated SNPs was not significantly associated with lipid-related traits. However, the RCT GRS, which includes SNPs in CETP and ABCA1 previously linked to blood lipids in the Mexican population, was significantly associated with both traits. CETP facilitates the exchange of cholesterol with TG), and ABCA1 promotes the return of cholesterol to the liver via HDL-C [36]. Dyslipidemia can disrupt this process by increasing circulating TG-rich lipoproteins and decreasing CETP activity, as seen in animal models of diet-induced dyslipidemia [37]. The complex effects of dyslipidemia on reverse cholesterol transport (RCT) in humans remain unclear, but genetic variation and diet-induced dyslipidemia may interact to influence RCT activity. Variants in CETP, such as rs1532624 and rs289714, are associated with reduced protein activity, while CETP-rs5882, a missense variant, is repeatedly associated with Alzheimer’s disease and macular degeneration risk [38,39]. The ABCA1-rs4149310 variant is specifically associated with HDL-C concentrations in Mexican, but not in European populations [8]. Since low HDL-C and high TG are common among Mexicans, variants in these genes might contribute to this phenotypic pattern. The ABCA1-rs9282541 variant primarily drives the association with HDL-C; removing it from the model makes this association non-significant. This variant decreases ABCA1 activity, resulting in smaller HDL-C particles. The SNPs studied in the GRS RCT pathway may interact with other unaccounted genetic or epigenetic factors, influencing the observed effects. Still, more work is needed in this area [34,35].

In younger populations, such as this college-age group, the clinical manifestations of cardiometabolic genetic risk are often subtle because environmental exposures are still limited, or the metabolic disease has not yet developed. Therefore, even small differences within the normal range may indicate early-stage expression of genetic liability that can widen with age [40,41,42,43,44]. A recent guideline-recommended cutoff point for TG levels notes the following categories: (1) <90 mg/dL as “Optimal”; (2) 90 ≤ TG < 130 mg/dL as “Early metabolic risk”; (3) 130 ≤ TG < 150 mg/dL as “Borderline metabolic risk”; and (4) TG ≥ 150 mg/dL as “Clinical hypertriglyceridemia” [40]. The prevalence in our cohort was 42% in the “Optimal” category and 33.8%, 7.9%, and 16% in the other categories, respectively. Thus, 58% of this population has sub-optimal TG values. As recently reported in larger population groups, even a small TG difference, sustained over long periods of the life cycle, may increase the risk of adult clinical dyslipidemia or atherosclerotic disease [40,45]. That is what we observed in the high- and low-risk genetic associations between the GRS RCT and the combined GRS (Supplemental Table S3).

Some of the genetic associations observed were only significant in males. However, we were underpowered to explore gene–sex interactions further at this time. We expect to secure resources to expand DNA extraction and genotyping for this cohort. The literature reports that males consistently have higher TG levels and a more atherogenic lipid profile than females, particularly in younger age groups and cohorts [41,46,47,48,49]. There are several possible biological explanations: (a) women secrete more TG-rich VLDL particles but have higher clearance rates, leading to lower circulating TG levels; (b) testosterone levels are associated with higher TG levels; (c) men have greater visceral fat storage, contributing to higher TG levels and increased metabolic risk. We do not have data from this cohort to evaluate any of these explanations. However, we will follow up with any other available phenotypic reports.

This study has some limitations. It did not account for lifestyle factors like diet, physical activity, and smoking, which are known to affect dyslipidemia. The sample size is relatively small compared to other genetic studies [34]. Nonetheless, given the limited research on young Mexicans, this study provides valuable insights into genetic correlations among different populations. Prior research, including our data, indicates that the frequency of risk alleles (MAF) varies across the Mexican population, emphasizing the need for further studies on the genetic factors contributing to dyslipidemia in this high-risk group [25,27,29,31,50,51]. An additional strength of this study is its focus on young adults who are at risk but have not yet developed dyslipidemia. Given the limited research on this age group, early detection of metabolic diseases to encourage lifestyle and behavioral changes could be more effective when introduced early rather than later.

## 5. Conclusions

In summary, findings from this study suggest that this group of emerging adults is at increased risk of dyslipidemia due to genetic variants in pathways related to lipoprotein metabolism. Several individual SNP associations were identified with TG and HDL-C blood concentrations. The SNPs included in this research had been previously associated with dyslipidemia, but most have not been investigated in young Mexican populations before.

Future research should explore the impact of genetic admixture, sex-specific factors, and lifestyle influences on genetic susceptibility to dyslipidemia in this population.

## Figures and Tables

**Table 1:** Single-nucleotide polymorphisms (SNPs) included in the genetic risk score.

Gene ^1^	Locus	Protein Function	SNP	Function of Variant	Risk Allele	MAF Global	MAF MXL	MAF UP-A
Reverse Cholesterol Transport Pathway (RCT)								
CETP	16q13	Facilitates the exchange of cholesterol esters for TGs between lipoproteins in circulation	rs1532624	Intron variant, associated with low CETP activity	A	0.31	0.35	0.39
			rs289714	Intron variant, associated with low CETP activity	C	0.29	0.24	0.35
			rs5882	Missense variant	G	0.37	0.44	0.44
ABCA1	9q31.1	HDL-C-bound protein that transports intracellular cholesterol to HDL-C	rs4149310	Intron variant, associated with decreased HDL-C	A	0.46	0.35	0.38
			rs9282541	Missense variant	T	0.01	0.07	0.10
Intergenic locus	20p7	Unknown	rs805743	Identified in Mexican GWAS	C	0.35	0.33	0.30
Cellular Lipid Uptake Pathway (CLU)								
ANGPTL3	1p31.3	Hepatokine that inhibits LPL and increases TG and HDL-C concentrations	rs10889337	Intron variant, histone signature enhancer	A	0.41	0.34	0.43
ANGPTL4	19p13.2	PPAR target, dissociates LPL monomer	rs1044250	Missense variant	T	0.31	0.40	0.40
			rs2278236	Intron variant, associated with decreased HDL-C	C	0.49	0.49	0.48
			rs7255436	Intron variant, recently identified in lipid loci associated with GWAS	A	0.49	0.49	0.49
CD36	7q21.11	Scavenger receptor, binds to oxidized LDL and LCFA.	Rs1527483	An intron variant, may influence dietary fat intake	T	0.10	0.08	0.12
			rs10499859	Upstream transcript variant	G	0.35	0.50	0.48
LPL	8p21.3	Hydrolyzes TG to allow fatty acids from lipoproteins into circulation	rs12678919	Intron variant, associated with elevated HDL-C	G	0.05	0.05	0.05
Lipoprotein Formation Pathway (LPF)								
GCKR	2p23.3	Inhibits glucokinase	rs1260326	Missense variant	T	0.38	0.35	0.31
LIPC	15q21.3	Hepatic triglyceride lipase, also involved in lipoprotein uptake	rs1800588	Intron variant, at promotor region, associated with lower LIPC activity	T	0.29	0.49	0.41
MLXIPL	7q11.23	Promotes TG synthesis by binding to carbohydrate response element motifs	rs2286276	Associated with elevated TG in both Mexican and European populations	G	0.27	0.35	0.40
PPARG	3p.25.2	Nuclear receptor, regulator of adipocyte differentiation	rs1801282	Missense variant	G	0.11	0.13	0.12
			rs12639162	Upstream transcript variant	G	0.43	0.49	0.46

^1^ CETP: Cholesterol Esterase Transfer Protein; ABCA1: ATP-binding Cassette Transporter member 1, subfamily A; ANGPTL3: Angiopoietin-like protein 3; ANGPTL4: Angiopoietin-like protein 4; CD36: Cluster of differentiation 36; LPL: lipoprotein lipase; GCKR: Glucokinase Regulator; LIPC: Hepatic lipase; MLXIPL: MLX-like interacting like protein; PPARG: Peroxisome Proliferator-Activator Receptor Gamma; MAF: minor allele frequency; MXL: Mexicans living in Los Angeles (*n* = 107). Global MAF obtained from the 1000 Genomes cohort. UP-A, MAF in the UP-AMIGOS Cohort (*n* = 580).

**Table 2:** Demographic and dietary data for the UP-AMIGOS Cohort ^1^.

Characteristics	Total (*n* = 580)	Males (*n* = 265)	Females (*n* = 315)	*p*-Value
Age (years)	18.9 ± 0.05	18.9 ± 0.08	18.8 ± 0.07	0.58
BMI (kg/m^2^)	23.6 ± 0.18	24.2 ± 0.28 *	23.5 ± 0.24 *	0.01
%OW, OB	17, 13	24, 10	15, 8	
TC (mg/dL)	170.4 ± 1.34	168.7 ± 1.98	171.9 ± 1.81	0.27
TG (mg/dL)	108.5 ± 2.07	109.7 ± 2.91	105.7 ± 2.93	0.60
HDL-C (mg/dL)	49.6 ± 0.48	47.6 ± 0.70 *	51.6 ± 0.65 *	<0.01

^1^ Data are presented as mean ± standard error. BMI: Body Mass Index; TC: Total Cholesterol; TG: Triglycerides; HDL-C: High-density lipoprotein Cholesterol. Student’s *t*-test determined differences between males and females. * Indicates significance at *p* < 0.05.

**Table 3:** Associations between genetic risk scores (GRS) and TG and HDL-C concentrations in the UP-AMIGOS cohort ^1^.

Pathway	Phenotype	Additive					Weighted				
		Model *p*-Value	% Variability Explained by the Model	GRS *p*-Value	% Variability Explained by GRS	β (95% CI)	Model *p*-Value	% Variability Explained by the Model	GRS *p*-Value	% Variability Explained by GRS	β (95% CI)
***RCT***	TG	0.0037	4.95 *	0.0275 *	3.43	3.59 (1.10, 6.04) *	0.0771	NC	0.1487	NC	NC
***CLU***	TG	0.0113	3.97	0.0528	NC	NC	0.0023	16.74 *	0.0072 *	14.44	0.83 (0.05, 1.61) *
***LPF***	TG	0.1276	NC	0.8239	NC	NC	0.0285	18.22	0.0656	NC	NC
***Total***	TG	0.0017	7.08 *	0.0108 *	6.07	2.35 (0.65, 4.04) *	0.5951	NC	0.6969	NC	NC
***Top Hits***	TG	0.011	3.41 *	0.0545	NC	NC	0.6829	2.31	0.8341	NC	NC
***RCT***	HDL-C	0.0079	5.59	0.2622	NC	NC	0.0004	31.45	0.0028 *	28.21	0.10 (−0.34, 0.53)
***CLU***	HDL-C	0.0624	NC	0.9949	NC	NC	0.0518	NC	0.1835	NC	NC
***LPF***	HDL-C	0.0545	NC	0.2592	NC	NC	0.6519	NC	0.9104	NC	NC
***Total***	HDL-C	0.0532	NC	0.4201	NC	NC	0.0005	4.02	0.0342 *	0.91	0.20 (0.02, 0.39) *
***Top Hits***	HDL-C	0.0036	2.70	0.3454	NC	NC	0.0160	9.17	0.2171	NC	NC

Additive GRS were calculated as the sum of the risk alleles present in an individual for variants in each pathway. Weighted GRS were calculated as the individual SNPs weighted by their β-coefficients from linear regression analyses. RCT = Reverse Cholesterol Transport; CLU = Cellular Lipid Uptake; LPF = Lipoprotein Formation; TG = Triglycerides; HDL-C = High-Density Lipoprotein Cholesterol. * Values indicate statistical significance at *p* < 0.05. If the GRS was not significantly associated with the phenotype, the percent (%) variability explained by GRS was not calculated (NC). ^1^ Models adjusted by age and sex.

**Table 4:** Associations between GRS and TG and HDL-C concentrations in the UP-AMIGOS cohort, including body mass index (BMI) in the statistical model ^1^.

Pathway	Phenotype	Additive					Weighted				
		Model *p*-Value	% Variability Explained by the Model	GRS *p*-Value	% Variability Explained by GRS	β (95% CI)	Model *p*-Value	% Variability Explained by the Model	GRS *p*-Value	% Variability Explained by GRS	β (95% CI)
***RCT***	TG	<0.0001	17.9	0.0135 *	3.29	3.85 (1.52, 6.15) *	<0.0001	31.58	0.2893	NC	NC
***CLU***	TG	<0.0001	16.31	0.0898	NC	NC	<0.0001	26.51	0.0051	12.65	0.44 (0.01, 0.87) *
***LPF***	TG	<0.0001	14.82	0.8239	NC	NC	<0.0001	23.98	0.219	NC	NC
***Total***	TG	<0.0001	18.07	0.0165	4.89	2.01 (0.41, 3.60) *	<0.0001	12.33	0.6969	NC	NC
***Top Hits***	TG	<0.0001	17.3	0.3669	NC	NC	0.0007	33.95	0.7272	NC	NC
***RCT***	HDL-C	<0.0001	14.84	0.0842	NC	NC	<0.0001	33.01	0.0061 *	24.74	0.11 (−0.30, 0.52)
***CLU***	HDL-C	<0.0001	11.27	0.9882	NC	NC	<0.0001	35.43	0.1089	NC	NC
***LPF***	HDL-C	<0.0001	14.7	0.2592	NC	NC	0.0016	25.98	0.7762	NC	NC
***Total***	HDL-C	<0.0001	14.58	0.4201	NC	NC	<0.0001	12.74	0.0186 *	1.01	0.22 (0.04, 0.39) *
***Top Hits***	HDL-C	<0.0001	14.11	0.7272	NC	NC	<0.0001	18.47	0.1359	NC	NC

Additive GRS were calculated as the sum of the risk alleles present in an individual for variants in each pathway. Weighted GRS were calculated as the individual SNPs weighted by their β-coefficients from linear regression analyses. RCT = Reverse Cholesterol Transport; CLU = Cellular Lipid Uptake; LPF = Lipoprotein Formation; TG = Triglycerides; HDL-C = High-Density Lipoprotein Cholesterol. * Values indicate statistical significance at *p* < 0.05. If the GRS was not significantly associated with the phenotype, the percent (%) variability explained by GRS was not calculated (NC). ^1^ Models adjusted by age, sex, and BMI.

## Data Availability

The data that support the findings of this study are available on request from the corresponding author. The data are not publicly available due to privacy and ethical restrictions.

## Supplementary Materials

The following supporting information can be downloaded at: https://www.mdpi.com/article/10.3390/nu18060979/s1, Table S1: Individual single-nucleotide polymorphism (SNP) associations for triglyceride (TG) concentrations in the UP-AMIGOS Cohort. Table S2: Individual single-nucleotide polymorphism (SNP) associations for high-density lipoprotein cholesterol (HDL-C) concentrations in the UP-AMIGOS Cohort. Table S3: Average genetic risk scores (GRSs) for the UP-AMIGOS Cohort. Table S4: Mean triglyceride (TG) and high-density lipoprotein cholesterol (HDL-C) concentrations by category of additive genetic risk. Table S5: Β-coefficients for weighted genetic risk score (GRS).

## Abbreviations

The following abbreviations are used in this manuscript:

HDL-C	High-Density Lipoprotein Cholesterol
TG	Triglyceride
RCT	Reverse Cholesterol Transport
CLU	Cellular Lipid Uptake
LPF	Lipoprotein Formation
CETP	Cholesterol Esterase Transfer Protein
ABCA1	ATP-Binding Cassette Transporter Member 1, Subfamily A
ANGPTL3/4	Angiopoietin-Like Protein 3/4
CD36	Cluster of Differentiation 36
LPL	Lipoprotein Lipase
GCKR	Glucokinase Regulator
LIPC	Hepatic Lipase
MLXIPL	MLX-Like Interacting-Like Protein
PPARG	Peroxisome Proliferator Activator Gamma
SNP	Single-Nucleotide Polymorphism
