# Supplementary material for: Pathway-Based Genetic Risk Scores Are Associated with Blood Lipids Among Young Mexican Adults
Source: Nutrients. 2026 Mar 19;18(6):979. doi: 10.3390/nu18060979 (PMC13029166; doi:10.3390/nu18060979)
Supplement: Supplementary file 1 [file nutrients-18-00979-s001.zip › nutrients-4071114-supplementary.pdf]

Table S1. Individual single-nucleotide polymorphism (SNP) associations for triglyceride (TG) concentrations in the UP-AMIGOS cohort.

SNP	Mode of Inheritance					
	Genotypic		Dominant		Recessive	
	Model P-value	SNP P-value	Model P-value	SNP P-value	Model P-value	SNP P-value
rs1532624	**0.0376**	**0.0442**	**0.0292**	0.2951	**0.0313**	0.1343
rs289714	**0.0333**	0.1783	**0.0277**	0.1669	**0.0284**	0.7949
rs5882	**0.0237**	**0.0488**	**0.0285**	0.2727	**0.0296**	0.3318
rs4149310	**0.0386**	0.0518	**0.0310**	0.0941	**0.0311**	0.1449
rs9282541	**0.0316**	0.9309	**0.0284**	0.7758	**0.0291**	0.4572
rs805743	**0.0327**	0.5459	**0.0283**	0.5009	**0.0305**	0.2013
rs10889337	**0.0318**	0.7768	**0.0286**	0.4303	**0.0288**	0.5381
rs1044250	**0.0361**	0.2188	**0.0305**	0.2339	**0.0286**	0.6694
rs2278236	**0.0378**	0.0644	**0.0306**	0.1218	**0.0286**	0.6461
rs7255436	**0.0346**	0.1965	**0.0271**	0.6022	**0.0293**	0.3954
rs1527483	**0.0320**	0.8840	**0.0282**	0.8827	**0.0299**	0.2681
rs10499859	**0.0339**	0.3429	**0.0284**	0.3532	**0.0301**	0.2521
rs12678919	**0.0318**	0.6547	**0.0271**	0.8346	**0.0292**	0.4070
rs1260326	**0.0331**	0.2986	**0.0270**	0.5035	**0.0298**	0.2921
rs1800588	**0.0368**	0.0515	**0.0262**	0.3208	**0.0326**	0.0693
rs2286276	**0.0484**	**0.0023**	**0.0470**	**0.0008**	**0.0358**	**0.0308**
rs1801282	**0.0280**	0.9729	**0.0278**	0.8490	**0.0284**	0.8870
rs12639162	**0.0354**	0.0749	**0.0329**	0.0616	**0.0312**	0.1403

Associations between TG concentrations and SNPs were determined by general linear models adjusted for age and sex. The Bonferroni correction was applied for multiple comparisons. In **bold**, statistical significance determined at $P<0.05$.

Table S2. Individual single nucleotide polymorphism (SNP) associations for high-density lipoprotein cholesterol (HDL-C) concentrations in the UP-AMIGOS cohort.

SNP	Mode of Inheritance					
	Genotypic		Dominant		Recessive	
	Model P-value	SNP P-value	Model P-value	SNP P-value	Model P-value	SNP P-value
rs1532624	**0.0498**	0.2164	**0.0384**	0.3042	**0.0382**	0.3336
rs289714	0.0514	0.0901	**0.0403**	0.1102	**0.0410**	0.0803
rs5882	**0.0497**	0.3597	**0.0398**	0.1405	**0.0376**	0.4882
rs4149310	**0.0498**	0.2164	**0.0384**	0.3042	**0.0382**	0.3336
rs9282541	0.0606	**0.0157**	**0.0392**	0.1885	**0.0442**	**0.0181**
rs805743	**0.0471**	0.6520	**0.0381**	0.3489	**0.0371**	0.8414
rs10889337	**0.0470**	0.5850	**0.0377**	0.4497	**0.0382**	0.3451
rs1044250	**0.0472**	0.5465	**0.0375**	0.5325	**0.0382**	0.3209
rs2278236	**0.0482**	0.4664	**0.0378**	0.4460	**0.0388**	0.2328
rs7255436	**0.0481**	0.4064	**0.0391**	0.2028	**0.0373**	0.6249
rs1527483	**0.0487**	0.6191	**0.0371**	0.5827	**0.0379**	0.4169
rs10499859	**0.0499**	0.1878	**0.0377**	0.4640	**0.0406**	0.0943
rs12678919	**0.0494**	0.2150	**0.0387**	0.2584	**0.0388**	0.2442
rs1260326	**0.0476**	0.5477	**0.0370**	0.9479	**0.0380**	0.3926
rs1800588	0.0507	0.0926	**0.0419**	0.0515	**0.0392**	0.1947
rs2286276	**0.0469**	0.6055	**0.0491**	0.4843	**0.0372**	0.6743
rs1801282	**0.0462**	0.9257	**0.0373**	0.6573	**0.0372**	0.6809
rs12639162	**0.0475**	0.6784	**0.0371**	0.7935	**0.0377**	0.4653

Associations between HDL-C concentrations and single-nucleotide polymorphisms (SNPs) were determined by general linear models adjusted for age and sex. The Bonferroni correction was applied for multiple comparisons. In **bold**, statistical significance determined at $P<0.05$.

Table S3. Average Genetic Risk Scores (GRS) for UP-AMIGOS Cohort.

		Additive				Weighted			
**Phenotype**	**Pathway**	Mean score (Total)	Median	Mean score (Males)	Mean score (Females)	Mean score (Total)	Median	Mean score (Males)	Mean score (Females)
TG	Reverse Cholesterol Transport	4.4 ± 0.1	4	4.5 ± 0.2	4.3 ± 0.1	0.1 ± 0.1	0.1	0.1 ± 0.0	0.1 ± 0.0
HDL-C		3.8 ± 0.1	4	3.7 ± 0.1	3.9 ± 0.1	1.2 ± 0.1	1.4	1.0 ± 0.2	1.4 ± 0.1
TG	Cellular Lipid Uptake	5.5 ± 0.1	5	5.5 ± 0.1	5.6 ± 0.1	0.1 ± 0.0	0.1	0.1 ± 0.0	0.2 ± 0.0
HDL-C		5.3 ± 0.1	5	5.2 ± 0.0	5.3 ± 0.1	-9.6 ± 0.3	-11.0	-9.5 ± 0.3	-9.7 ± 0.3
TG	Lipoprotein Formation	5.4 ± 0.1	5	5.5 ± 0.1	5.4 ± 0.1	-0.1 ± 0.0	-0.01	-0.1 ± 0.1	-0.1 ± 0.1
HDL-C		5.4 ± 0.1	5	5.5 ± 0.1	5.4 ± 0.1	-2.6 ± 0.1	-2.3	-2.7 ± 0.1	-2.6 ± 0.1
TG	Combined GRS	15.3 ± 0.2	15	15.5 ± 0.2	15.2 ± 0.2	0.2 ± 0.1	0.1	0.1 ± 0.0	0.2 ± 0.0
HDL-C		14.6 ± 0.2	15	14.5 ± 0.2	14.5 ± 0.2	-11.0 ± 0.3	-11.9	-11.2 ± 0.4	-10.9 ± 0.3

Additive genetic risk scores were calculated as the sum of the risk alleles present in an individual for variants within each pathway. Weighted genetic risk scores were determined by multiplying each SNP by its β-coefficient derived from linear regression analyses. Student's t-test was used to compare males and females, with * indicating significance at P<0.05. Data are shown as mean ± standard error.

Table S4. Mean triglyceride (TG) and high-density lipoprotein cholesterol (HDL-C) concentrations by category of additive genetic risk (median number of risk alleles).

**Pathway**	**Risk Category** (TG/HDL)	TG (mg/dL)	P-Value	HDL-C (mg/dL)	P-Value
Reverse Cholesterol Transport	Low <5/4	103.1 ± 2.5 (n=328)	**0.002**	50.4 ± 0.7 (n=278)	0.082
	High >5.1/4.1	115.7 ± 3.6 (n=252)		48.7 ± 0.7 (n=302)	
Cellular Lipid Uptake	Low <4/5	107.4 ± 2.3 (n=217)	0.419	49.6 ± 0.6 (n=389)	0.685
	High >4.1/5.1	111.3 ± 5.0 (n=363)		49.5 ± 0.8 (n=191)	
Lipoprotein Formation	Low <5	108.0 ± 2.7 (n=243)	0.622	49.3 ± 0.6 (n=243)	0.069
	High >5.1	108.4 ± 3.3 (n=337)		50.1 ± 0.8 (n=337)	
Combined GRS	Low <15	103.7 ± 2.5 (n=267)	**0.003**	49.9 ± 0.6 (n=308)	0.782
	High >15.1	114.7 ± 3.5 (n=313)		49.2 ± 0.7 (n=272)	

Data are shown as mean ± standard error. High- and low-risk genetic groups were defined based on the sample median of risk alleles for each pathway. The means are adjusted for age, sex, and Body Mass Index.

Table S5. β-coefficients for weighted Genetic Risk Score (GRS).

Pathway	Phenotype	*Gene*-SNP	β coefficient genotypic			β coefficient dominant		β coefficient recessive	
			**0**	**1**	**2**	**0**	**1**	**0**	**1**
Reverse Cholesterol Transport	TG	*LOC*-rs805743	0.0096	0.0217	0	0.0153	0	0.0165	0
	TG	*CETP*-rs1532624	-0.0336	-0.0084	0	-0.0186	0	-0.019	0
	TG	*CETP*-rs289714	-0.0107	0.0212	0	-0.0188	0	0.0053	0
	TG	*CETP*-rs5882	-0.0282	-0.0124	0	-0.0189	0	-0.0136	0
	TG	*ABCA1*-rs4149310	0.0427	0.0202	0	-0.0337	0	-0.0197	0
Cellular Lipid Uptake	TG	*ANGPTL3*-rs10889337	-0.0183	-0.0105	0	-0.0127	0	-0.0078	0
	TG	*ANGPTL4*-rs2278236	0.0091	0.0303	0	0.0232	0	-0.0072	0
	TG	*CD36*-rs10499859	0.0294	0.0219	0	-0.0143	0	-0.0196	0
	TG	*LPL*-rs12678919	0.0983	0.099	0	-0.0045	0	-0.0948	0
	TG	*LPL*-rs13702	-0.0066	0.0168	0	0.0089	0	-0.0124	0
Lipoprotein Formation and Transport	TG	*GCKR*-rs1260326	-0.0266	0.0048	0	-0.0151	0	-0.0148	0
	TG	*LIPC*-rs1800588	0	-0.0318	0	-0.0185	0	0.0259	0
	TG	*MLXIPL*-rs2286276	-0.0773	-0.0407	0	-0.0479	0	-0.0654	0
	TG	*PPARG*-rs1801282	0.0091	0.0041	0	-0.0054	0	-0.0048	0
	TG	*PPARG*-rs12639162	-0.0423	-0.0232	0	-0.0303	0	-0.0206	0
Reverse Cholesterol Transport	HDL-C	*LOC*-rs805743	1.405	1.523	0	1.46	0	0.2264	0
	HDL-C	*CETP*-rs1532624	-2.458	-0.8764	0	1.775	0	1.379	0
	HDL-C	*CETP*-rs289714	-3.005	-1.457	0	2.246	0	1.635	0
	HDL-C	*CETP*-rs5882	-1.69	-0.33	0	1.459	0	0.7686	0
	HDL-C	*ABCA1*-rs9282541	3.83	0.066	0	3.19	0	2.236	0
Cellular Lipid Uptake	HDL-C	*ANGPTL3*-rs10889337	-1.515	-0.6912	0	1.093	0	0.8115	0
	HDL-C	*ANGPTL4*-rs2278236	1.667	0.4596	0	0.8646	0	1.267	0
	HDL-C	*CD36*-rs10499859	-2.09	-1.96	0	0.7418	0	1.902	0
	HDL-C	*LPL*-rs12678919	-8.814	6.833	0	2.344	0	8.693	0
	HDL-C	*LPL*-rs13702	-1.482	-1.448	0	-1.459	0	-0.5835	0
Lipoprotein Formation and Transport	HDL-C	*GCKR*-rs1260326	0.4174	-0.696	0	-0.1212	0	0.8299	0
	HDL-C	*LIPC*-rs1800588	-2.92	-0.8	0	2.455	0	1.199	0
	HDL-C	*MLXIPL*-rs2286276	0.6286	-1.228	0	-0.8656	0	0.8218	0
	HDL-C	*PPARG*-rs1801282	-1.509	-1.347	0	0.2527	0	1.404	0
	HDL-C	*PPARG*-rs12639162	0.6563	-0.0929	0	0.1839	0	0.6288	0

β coefficients were obtained from general linear models adjusted for age, sex, and BMI. *Indicates if a β coefficient was significantly different from 0 at $P<0.05$.
